# Perception Methods for Adverse Weather Based on Vehicle Infrastructure Cooperation System: A Review

**DOI:** 10.3390/s24020374

**Published:** 2024-01-08

**Authors:** Jizhao Wang, Zhizhou Wu, Yunyi Liang, Jinjun Tang, Huimiao Chen

**Affiliations:** 1 School of Mechanical Engineering, Xinjiang University, Urumqi 830017, China; jizhaowang@stu.xju.edu.cn; 2 College of Transportation Engineering, Tongji University, Shanghai 201804, China; 3 School of Traffic & Transportation Engineering, Central South University, Changsha 410075, China; 22022049@csu.edu.cn (Y.L.); jinjuntang@csu.edu.cn (J.T.); 4 Tsinghua Laboratory of Brain and Intelligence, Tsinghua University, Beijing 100084, China

**Keywords:** ICV, autonomous driving, adverse weather conditions, data preprocessing, multi-sensor information fusion, vehicle–infrastructure cooperation perception

## Abstract

Environment perception plays a crucial role in autonomous driving technology. However, various factors such as adverse weather conditions and limitations in sensing equipment contribute to low perception accuracy and a restricted field of view. As a result, intelligent connected vehicles (ICVs) are currently only capable of achieving autonomous driving in specific scenarios. This paper conducts an analysis of the current studies on image or point cloud processing and cooperative perception, and summarizes three key aspects: data pre-processing methods, multi-sensor data fusion methods, and vehicle–infrastructure cooperative perception methods. Data pre-processing methods summarize the processing of point cloud data and image data in snow, rain and fog. Multi-sensor data fusion methods analyze the studies on image fusion, point cloud fusion and image-point cloud fusion. Because communication channel resources are limited, the vehicle–infrastructure cooperative perception methods discuss the fusion and sharing strategies for cooperative perception information to expand the range of perception for ICVs and achieve an optimal distribution of perception information. Finally, according to the analysis of the existing studies, the paper proposes future research directions for cooperative perception in adverse weather conditions.

## 1. Introduction

Intelligent connected vehicles (ICVs) represent the next generation of vehicles, incorporating chips, big data, and artificial intelligence, which have a potential to greatly enhance traffic operation efficiency, while reducing energy consumption and carbon emissions. They utilize sensors to gather environmental information in their surroundings [1]. And the vehicle’s behavior planning system and dynamic control system then uses these perception results for tasks such as trajectory prediction [2], lane-changing [3], and vehicle power control [4]. The seamless integration of perception, planning and control methods on ICVs ensures that ICVs realize safe autonomous driving on the road [5]. It is evident that environment perception is one of the most important components within the autonomous driving system [6], and provides essential perception information, which assists the decision system and control system to make informed decisions and take appropriate actions.

Despite these advancements, intricate components within a transportation system pose challenges for sensors on an individual ICV within a limited perception range [7]. For instance, when an ICV is in motion or parked alongside buses or trucks, it may be unable to capture pedestrian and vehicle information on the opposite side of these taller vehicles. The lack of information in perception-blind areas prevents ICVs executing timely emergency braking measures, and results in potential traffic accidents. Adverse weather conditions [8], such as fog, snow, rain, and sunlight, often degrade the color and texture in images, and disrupt the distribution structure of the LiDAR points with noisy data. Utilizing the inaccurate data for environment perception can lead to misjudgments or malfunctions in the vehicle perception system [9]. Thereby, many ICVs currently only achieve partially autonomous driving functions, such as lane-keeping, automatic parking, intelligent speed limitation, autobrake, and adaptive cruise control. According to the taxonomy scale established by The Society of Automotive Engineers (SAE) and the Standardization Administration of China (SAC), these vehicles belong to Level 2 vehicles (L2) [10,11]. In the level, the autonomous driving system assists drivers in dynamic driving tasks, with drivers remaining the primary driving subject. Additionally, conditionally automated driving vehicles (Level 3 vehicle, L3) and highly automated driving vehicles (Level 4 vehicle, L4) operate in specific areas, such as ports, mines, highways and urban roads. These vehicles from L3 have depended on autonomous driving systems to perform dynamic driving tasks under the predefined operating conditions, instead of human driving involvement. Table 1 shows the taxonomy of intelligent connected vehicles [11].

To mitigate the negative impacts of adverse weather conditions in raw perception data, many studies have employed Bayesian estimation [12], neighborhood filtering [13], and Voxel filtering [14] to eliminate noisy data from raw LiDAR points in adverse weather conditions. Additionally, integrating multi-scale information related to semantic segmentation [15], depth [16] and color [17] into Gaussian low-pass filters [18], bilateral filters [19] and deep learning networks [20] has been explored for image enhancement. But these preprocessing methods in dense fog, heavy rain and heavy snow have poor results. It is necessary to further investigate the preprocessing methods for challenging weathers to enhance the robustness of ICV perception systems in adverse environments. Other research papers have endeavored to mitigate the uncertainty and vulnerability of a single sensing system by integrating information from multiple sources, including cameras, LiDAR, and millimeter-wave radar in adverse weather conditions. Techniques such as the Generative Adversarial Network [21], Multi-view 3D Networks [22], and RadarNet learning architecture [23] have been employed to fuse data from various sensors on an intelligent perception agent, enhancing perception precision in specific scenarios. However, when detected objects are far away from sensors, the perception accuracy declines greatly. This is the reason for the loss of texture and color features in images and the scarcity of point cloud data for distant objects.

The most recent advancements in deep learning methods have gained more accurate calculation results. However, the technical bottlenecks and limitations inherent in achieving fully automated vehicles (L5) must be addressed, and the frameworks of ICVs and vehicle to everything (V2X) systems need to be optimized [24] to provide additional environment information and computing resources from roadside infrastructures [25]. A vehicle–infrastructure cooperation system (VICS) is defined as the coupling and collaboration of four key elements in the road traffic system: human, vehicle, road, environment. It integrates the Internet of Everything, artificial intelligence, mobile internet, 5th-Generation Mobile Communication (5G), and edge computing to acquire additional information from cameras, radar, LiDAR and other sensors installed on vehicles and roadside poles. The synchronous perceptions of vehicles and roadside terminals significantly reduce the cost of data-driven algorithms on intelligent perception units and aid in predicting the intentions of traffic participants [26,27]. It becomes a new technology to provide driving assistance, and promotes the development of fully autonomous driving. America, the European Union, Japan and China have conducted extensive research efforts to develop and implement VICS since the last century.

In the early 1970s, General Motors Corporation [28] in America carried out experimental research on highway automation under the background of ITS research. It was the beginning of VICS. The University of California, Berkeley [29] carried out the PATH project, utilizing communication technology to connect On-Board Units (OBU) with Road-Side Units (RSU). And they constructed the Intelligent Vehicle/Highway System (IVHS) to assist drivers to make driving decisions. A dedicated radio service called Dedicated Short Range Communications (DSRC) was introduced in ITS, utilizing communications with IEEE 802.11p and a 75 MHz band to enhance traffic safety and alleviate congestion in 1998. In 2003, researchers developed an intelligent vehicle equipped with a GPS module and a wireless communication module, utilizing communication technology to exchange collected data between the vehicle and traffic infrastructures [30]. An international standard for VICS was published in 2016 [31]. According to the previous technical exploration and the experience of road intelligent upgrading, test sites such as the Virginia Smart Road test site, American Center for Mobility, and GoMentum Station were built to further test VICS capabilities in the past two decades.

In order to develop intelligent vehicles, the European Union proposed the Prometheus Project for the European automotive industry [32]. In 2003, they proposed the e-Safety project, including a vehicle autonomous safety system and a vehicle–infrastructure cooperation system [33]. They analyzed the information from vehicles and roadside devices and generated a safety management scheme for the road traffic system, and developed vehicle autonomous safety devices. And then the Car2Car league, comprising some vehicle electronic manufacturers and other vehicle manufacturers, carried out several experiments on vehicle infrastructure cooperation systems and applicated the technology in real traffic scenarios [34]. The European Commission established a Cellular Vehicle-to-Everything (C-V2X) communication network in 2016 to assess the autonomous driving function of multiple trucks traveling in a convoy [35]. A European digitalization plan in EUROPE2020 and VICS test sites like Cooperative ITS Corridor were set up to support those studies on autonomous driving technology and cooperative perception methods.

Japan proposed the Super Smart Vehicle System (SSVS) in 1988, utilizing advanced electronic technology to enhance road intelligence and vehicle safety [36]. The subsequent Smart-way project under the principle of VICS aimed to integrate ITS functions into OBU, achieving the information communication between the Smart-way and vehicles. The ITS Spot project, under the background of the Smart-way project, installed 1600 roadside units on roads and an integrated vehicle–infrastructure communication platform with on-board units to test the function of VICS [37]. Based on the sharing of traffic big data, the ETC 2.0 project and JASI intelligent vehicle test base were proposed to support studies on automatic driving, including real-time location acquisition and data processing [38].

China started to research the combination of drivers, vehicles and traffic equipment in 1995 [39]. At the same time, on-board equipment and roadside equipment in intelligent transportation systems had been developed and optimized. The first key technology research project on vehicle infrastructure cooperation, part of the ‘863 Program’, was set up in 2011 [40]. The project established a test and verification experiment system for intelligent vehicle–infrastructure cooperation, building several test sites in cities, such as Shanghai and Chongqing. In 2014, ICV successfully completed the test in various typical application scenarios with the assistance of VICS, including blind spot warnings and multi-vehicle cooperative lane changes. China introduced a new communication standard, Long Term Evolution-Vehicle to Everything (LTE-V2X), to replace DSRC. The LTE-V2X with a 5905–5925 MHz band had advantages in a wider communication coverage, higher reliability of signal transmission, and higher bandwidth capacity. In 2019, the first ICV test section on an expressway was built to provide multi-source environmental information for ICVs [41]. At present, China has around 73 million connected vehicles and 14.8 million ICVs with an Advanced Driving Assistance System (ADAS).

VICS utilizes wireless communication for interaction and information sharing among vehicles as well as between vehicles and infrastructures, which can assist high-level autonomous vehicles to operate safely. The development of VICS can be categorized into five stages, as shown in Table 2 [42]. It is noted that the service subject of VICS has shifted from ordinary vehicles to ICVs in the 4.0 era, due to the development of mobile communication technology. Utilizing vehicle–infrastructure cooperation may be a good approach to overcome the insufficient environmental perception information of ICV in extreme weather and object occlusion situations. It could reduce the production cost of ICVs by transferring part of the autonomous driving function and computing tasks from ICVs to the roadside infrastructures. The technology has the potential to prompt ICV to achieving fully autonomous driving (L5) and large-scale commercial application. In the process of information interaction, a cooperative perception method optimally utilizes these information resources from multi-sensors and multiple agents to accurately describe the perception space in adverse weather conditions and blind zones [43]. The method employs optimization algorithms to select optimal information, determine the optimal information compression, transmission and fusion mode within limited communication channels. Some studies have implemented confidence evaluation [44], and graph theory [45] to reduce information redundancy, while others have explored information compression [46], variable frequency communication, and special data formats to maximize the use of the limited communication bandwidth.

**Figure 1:**
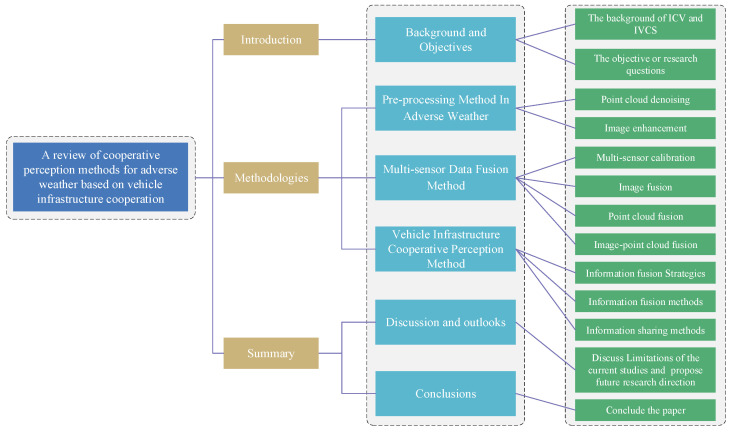
An overall diagram of the review on perception methods for adverse weather based on vehicle infrastructure cooperation system.

In summary, vehicle–infrastructure cooperation perception is a research hotspot in the field of intelligent transportation system (ITS) [27]. It serves as a promising approach to overcoming the technical bottlenecks and limitations of ICVs on single-vehicle environment perception, and enable ICVs to navigate in any complex circumstances effectively. The core components of vehicle–infrastructure cooperation perception include the processing of sensor data, multi-sensor information fusion, and the optimal selection and distribution of collaborative perception information. The structure of this paper is organized as shown in Figure 1. Section 2 summarizes the data pre-processing methods from two aspects in adverse weather conditions, focusing on LiDAR point processing and image processing. Section 3 analyzes the multi-sensor data fusion method in image fusion, point cloud fusion, and image-point cloud fusion. Section 4 summarizes the information fusion methods and sharing strategies for cooperative perception information. Section 5 discusses the current studies and proposes potential research directions for the future. Section 6 concludes this paper.

## 2. The Preprocessing Method in Adverse Weather Conditions

Adverse weather, such as rain, snow and fog, usually causes image blurring and color degradation, and disruption in the distribution of LiDAR points. To achieve accurate object detection on intelligent units or precise cooperation perception among multiple intelligent agents in adverse weather conditions, many studies have proposed various pre-processing methods to mitigate the adverse effects of adverse weather on original images or LiDAR points. This section mainly introduces the data pre-processing methods for LiDAR points and images in adverse weather conditions.

### 2.1. LiDAR Point Denoising in Adverse Weather Conditions

The methods for point cloud denoising can be summarized as statistical filtering, neighborhood filtering, projection filtering and voxel filtering.

Statistical filtering methods in many studies mainly include Bayesian estimation [12] or Principal Component Analysis (PCA) [47]. Schall et al. [48] used Maximum Likelihood Estimation (MLE) to construct a point cloud denoising method. The method used non-parametric kernel density estimation to estimate the probability of an effective point cloud on detection objects. Jenke et al. [12] applied a Bayesian statistics method to reduce the noisy data and generated smooth point cloud data. A nonlinear optimization method was employed to calculate the optimal distribution of point cloud data in real-world scenes. Rusu et al. [14] constructed a point cloud pre-processing library to remove the noisy points collected in adverse environments. The library included a voxel grid filter, a Statistical Outlier Removal (SOR) filter, and a Radius Outlier Removal (ROR) filter. Hu et al. [49] constructed an SOR method based on the principal of statistic distribution calculation. The method judged noisy data in point clouds by computing distance and standard deviation from the target point cloud data to K neighboring point clouds. Kurup et al. [50] proposed a Dynamic Statistical Outlier Removal (DSOR) method to process noisy data in point clouds collected in rain and snow weather. The DSOR network presented a straightforward framework and efficiently removed noisy data in less time. Luo et al. [51] regarded the distribution of a noisy point cloud as the distribution of a set of noise-free samples. They used a neural network to estimate the gradient of the log-probability function, using only noisy point clouds as input.

Neighborhood filtering methods mainly use bilateral filtering algorithm [52]. Schall et al. [13] proposed a LiDAR point denoising method based on the principle of image neighboring filter. The method constructed a similarity weighting function based on the results of the similarity between two points. Charron et al. [53] constructed a Dynamic Radius Outlier Removal (DROR) filter to mitigate negative factors. The filter not only preserved most details of environmental features, but also adapted to changes between point cloud density and detection distance. Wang et al. [54] proposed a Dynamic Distance-Intensity Outlier Removal (DDIOR) filtering method to denoise point cloud data. Roy et al. [55] utilized the wavelength and target reflectivity of an LiDAR sensor to construct an intensity filter, using an intensity threshold to remove snow noise in point cloud data. Park et al. [56] proposed a Low-intensity Outlier Removal (LIOR) method to remove noises. The method utilized the difference in intensity between snow noise points and detection object points at the same distance to judge neighborhood points within a specified search radius. Roriz et al. [57] integrated the LIOR method and DROR method to construct the Dynamic Light-intensity Outlier Removal (DIOR) method, further enhancing the accuracy of point cloud denoising.

Projection filtering methods use projection transformation [58] among multiple perspective views and different projection strategies [59] to eliminate noisy point cloud data. Charron et al. [53] converted sparse 3D LiDAR points to 2D data, and used a DROR filter to obtain smooth features of key points. It could avoid isolated LiDAR points being incorrectly removed on detection objects due to a lack of semantic information. Duan et al. [60] used the PCA algorithm to convert 3D point clouds into 2D point clouds, and then utilized Density-based Spatial Clustering of Applications with Noise (DBSCAN) to remove sparse point clouds. Heinzler et al. [61] merged a Convolutional Neural Network (CNN) and LiLaNet [62] to reduce point cloud noises in heavy rain or dense fog. The LiLaNet deep neural network architecture used virtual image projections of 3D point clouds to improve the efficient of a comprehensive semantic understanding. However, without the input of additional information, the fusion method cannot fully compensate for the visibility issues in foggy weather using point cloud data.

Voxel filtering methods need rasterized point cloud data. Balta et al. [63] proposed the Fast Cluster Statistical Outlier Removal (FCSOR) method to enhance the computational efficiency of point cloud denoising. The method included voxel sub-sampling and parallel computation. Shamsudin et al. [64] integrated K-Nearest Neighbor (KNN) and Support Vector Machine (SVM) to remove noisy data in foggy weather.

### 2.2. Image Enhancement in Adverse Weather Conditions

Image enhancement methods in many studies have used depth information, color information, semantic segmentation, prior knowledge [65], and deep learning approaches [20] to construct a variety of filtering methods. Tomasi et al. [18] used a Gaussian low-pass filter to remove noisy pixels in depth images. The method calculated the average weights of image depth information, and assigned these weights according to distance metrics. Chen et al. [66] used a bilateral filter and a maximum posteriori estimator to mark all pixels in images. And a region-growing algorithm was used to segment the image after the smoothing process. Shen et al. [17] proposed an image denoising method that integrated a bilateral filter with depth information. The depth prior information from the green, red and blue channels was obtained by computing pixel discontinuity values of colors and observation distances. Because different weather causes different negative factors on images, it is necessary to propose a variety of image enhancement methods.

For relatively static adverse weather, like fog, images tend to become blurry. And a lot of researches have proposed diverse methods to eliminate the noisy pixels of fog. He et al. [67] proposed a scene-prior image de-fogging algorithm, which used image depth information and transmission estimation to address fog-related issues. But the method needed manual interventions. Sim et al. [16] proposed a scene-prior method based on clear images and depth data. The method had a good visual treatment when images suffered from significant contrast and color distortion. Bijelic et al. [68] proposed an image enhancement method to deal with image scattering caused by fog, calculating light decay rates and detecting the scattered light signals. Yang [15] proposed a high-speed defogging network, 4DL1R-Net, to gain sharper images. The network integrated an adaptive-modified dark channel prior algorithm and a four-way adaptive L1 regularized filtering.

Relatively dynamic weather, such as rain and snow, not only blurred images, but also obscured critical object features in images. Rönnbäck et al. [19] designed a filter based on the bilateral filter framework. The filter could keep the edges smooth compared to traditional low-pass filters in deep image denoising. Kang et al. [69] firstly divided an image into low- and high-frequency layers according to the values of pixels and used a dictionary learning algorithm to remove rain from the high-frequency layer. Rajderkar [70] employed frequency space separation and color assumptions to construct a feature model for snow detection. Pei et al. [71] used the color and shape features of snowflakes to detect the location of snow in images. To distinguish raindrops from the background, Chen et al. [72] used a categorical dictionary method, while Luo [73] used discriminative sparse coding. Kim et al. [74] found that rain-line elliptic kernels exhibited specific angles in each pixel of images, and used an adaptive non-local mean filter to remove raindrops. Liu et al. [20] proposed a DesnowNet deep learning network to calculate the values of semi-transparency and used a residual generation module to remove snow from a single image. Li et al. [75] used the features of pixel-level space at different scales to construct a multi-scale snow removal network. Zhang [76] introduced the assumption of implicit image information into a Generative Adversarial Network (GAN). The network could separate texture features of rain and snow from image backgrounds. Zhang et al. [77] constructed Deep Dense Multi-Scale Networks (DDMSNet) to remove snow in images. And the networks integrated self-attentive mechanisms into semantic perception and geometric perception.

In other studies, redundancy information from the adjacent frames in videos was used to construct rain or snow models, with a frame difference method or low-rank matrix compensation method for image enhancement. Bossu et al. [78] employed a hybrid Gaussian model to separate foreground and background, and utilized a Histogram of Oriented Gradient (HOG) to detect and remove snow in the foreground segmentation. Xie [79] constructed a noisy-point removal algorithm to calculate grey values between adjacent frames, and used these grey values to detect rain and snow in videos. Kim et al. [74] used the feature of adjacent frame distortion to construct an optical flow method. The method could generate initial snowflake detection maps for snow removal. Tian et al. [80] used a global low-rank matrix decomposition method to separate snowflakes from backgrounds and foregrounds based on color and shape features of snow. The method used an absolute average deviation to obtain the low-rank structure of snow.

Overall, various studies have been conducted on image enhancement and point cloud denoising in adverse weather conditions. Good pre-processing results in mist, light rain and light snow were obtained. However, further research is required on processing methods in dense fog, heavy rain and heavy snow. Considering these studies that utilized redundancy information from adjacent frames to reduce negative factors in adverse weather conditions, it is worthwhile to explore the use of fusion data from multiple sensors on an ICV or fusion information among intelligent agents to eliminate the negative impact of adverse weather on images and LiDAR points.

## 3. Multi-Sensor Data Fusion Method

A multi-sensor data fusion method [9] is used to integrate perception information from multiple sensors, such as cameras, LiDAR, millimeter-wave radar and ultrasonic sensors. The fusion method aims to diminish uncertainty and enhance the robustness of environment perception using a single sensor in adverse weather conditions or complex traffic conditions. The application of this method extends to various domains, including object detection, automation, situation assessment, earth science and other fields. This section mainly summarizes and analyzes many methods related to multi-sensor calibration, image fusion, point cloud fusion, and image-point cloud fusion, which can improve the perception accuracy in ICVs.

### 3.1. Multi-Sensor Temporal and Spatial Calibration

The temporal and spatial calibration of multiple sensors is a fundamental process before the multi-source data fusion. Spatial calibration usually uses constant turn rates and a motion model based on acceleration to transform different sensor data into a unified coordinate system. Temporal calibration utilizes interpolation calculations to ensure temporal consistency among the data from multiple sources. Verma et al. [81] employed point cloud plane fitting and inverse perspective transformation to extract coordinates of the same feature in each sensor. A genetic optimization algorithm was then applied to calculate and optimize external parameters. Huang et al. [82] used inverse projection transformation to derive the transformation matrix of point cloud data and computed the minimized L1 norm distance. These parameters were employed to determine the optimal solution for point cloud data calibration within the field-of-view of ICVs. Zhang [83] considered timestamps of point cloud data as a crucial external parameter in calibration method. The method achieved the calibration of LiDAR and camera in both time and space.

### 3.2. Multi-Sensor Image Fusion Method

Images, with their high-resolution and detailed information such as object texture and color, have become the most important data for ICVs in perceiving environment. The results of multi-source image fusion can be used to estimate the spatial depth information, and compensate the perception of a single camera in adverse weather conditions. Fusion methods in some studies mainly involve compression and feature fusion of image data. Xiao et al. [84] employed deep learning to extract key perceptive feature of the vehicles in images and created a bird’s eye view (BEV) to merge these features from two visual sensors. Löhdefink et al. [85] proposed a lossy learning image compression method, utilizing an adversarial loss function [86] to integrate perception features from multiple sensors, and reduced data transformation overload within limited communicated bandwidth. Rubino et al. [87] and Cortés et al. [88] considered cross-image geometric constraints and object reidentification as a multi-sensor image fusion method. The method could deal with the challenge of object positioning in multi-view fusion. Lv et al. [21] utilized the GAN to integrate the static background and dynamic foreground based on the principles of pixel denoising and scene separation.

### 3.3. Multi-Sensor Point Cloud Fusion Method

Point cloud data from LiDAR have many advantages in target identification, distance measurement, and object positioning for environment perception. These data have high spatial resolution and are not influenced by transportation environments, such as sunlight. Chen et al. [89] used the principle of redundant point cloud sharing to construct a neural network model for object detection. The model made a good performance in multi-source point cloud fusion, particularly in the low-density scenarios. Ye et al. [90] utilized a state estimation framework and alignment method to match point cloud data from LiDAR sensors in different mounting positions. The method could represent the road from offline point cloud data accurately. Chen et al. [91] proposed a hybrid method for mering point cloud data based on two types of point cloud features obtained from voxel feature fusion and spatial feature fusion, respectively. Arnold et al. [92] introduced a point cloud hybrid fusion method based on a 3D object detection network. The method utilized the visibility and distribution characteristics of point cloud data at different distances.

### 3.4. Multi-Sensor Image-Point Cloud Fusion Method

The fusion of image data and point cloud data can compensate for the limitations of a single class of sensors. Cameras and LiDAR sensors, as discussed in Section 4.2 and Section 4.3, have distinct advantages in obtaining perceptive data. These fusion data are used to extract different types and dimensions of features. And it has become a crucial approach for enhancing environment perception accuracy, especially in adverse driving conditions. These image-point cloud fusion methods can be divided into Kalman filter-based fusion estimation methods, Bayesian-based distributed fusion methods, and neural network-based methods. Ji et al. [93] used radar detection to created Region of Interest (RoI) in an image. A neural network classified the RoI results, which were then used to fuse image and point cloud data. Wang et al. [94] integrated point cloud data and images by projecting the point cloud data into the image coordinate system. The method used a filter to filter out the background of detected objects, with automatic coordinate conversion and calibration. Chen et al. [22] integrated Multi-view 3D Networks (MV3D) and a PointFusion machine learning framework to aggregate feature vectors from point cloud data and images. Vora et al. [95] proposed a PointPainting method to merge point cloud data with the results of image semantic segmentation. Liang et al. [96] constructed a simplified two-stage detector with densely fused two-stream multi-sensor backbone networks. The detector applied RoI feature fusion, depth completion and ground estimation to incorporate the 3D detection boxes from LiDAR points and images. Yang et al. [23] utilized CNN networks to extract early features of sparse radar points and LiDAR points. And these features were used to perform feature fusion and object detection through the RadarNet learning architecture. Shah et al. [97] constructed an end-to-end approach (called LiRaNet) to merge the early features of a temporal sequence of radar data, LiDAR points and high-definition maps. The fusion features were then used to accurately predict vehicle trajectories. Satio et al. [98] integrated point cloud data and images by projecting LiDAR points into the coordinate system of the next frame image after temporal and spatial calibration of multiple sensors. The method could address the problem of sparse point clouds at long distances. Yang [99] proposed a multi-sensor 3D object detection backbone network with multi-scale feature fusion and feature-candidate region fusion. The method used voxel grids of point cloud data to match image features at the same timestamp and spatial coordinates.

### 3.5. Multi-Sensor Data Fusion Strategies

Data fusion is the basis of multi-sensor data processing in a single intelligent agent. Based on the types of data utilized (raw data, feature data, and detection results), multi-sensor data fusion strategies can be categorized into three levels: target-level fusion, feature-level fusion and data-level fusion.

**Figure 2:**
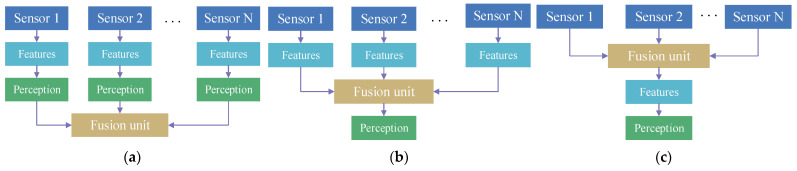
Three data fusion strategies on an intelligent agent. (**a**) shows the target-level fusion strategy; (**b**) shows the feature-level fusion strategy; (**c**) shows the data-level fusion strategy.

The target-level fusion strategy initially uses the raw perception data from each sensor to obtain object detection results, and generates a tracking list of detection objects. Subsequently, a fusion model is used to obtain an intact tracking list of multi-sensor detection results on a single intelligent agent. The model computes the correlation degree to evaluate each target tracking list from sensors, such as LiDAR, cameras and ultrasonic radars, and matches the tracking lists with higher correlation (Figure 2a). Because each sensor has its own object detection method to process heterogeneous data and gain detection boxes, the fusion strategy only integrates these detection boxes from different sensors. It has a fast speed of object fusion, but lacks sufficient information about detection objects. And the detection precision is usually very low, caused by the inaccurate perception results.

Feature-level fusion strategy firstly needs to eliminates the heterogeneity among images, LiDAR points and ultrasonic data through projection and transformation in the environment perceptive network. Subsequently, a feature extraction algorithm is employed to extracted object features based on these data. And a fusion algorithm is used to integrate these features into a single eigenvector (Figure 2b). Compared with target-level fusion, this fusion strategy reduces information loss and improve the detection precision relatively.

Data-level fusion strategy directly uses a multi-source fusion method to integrate raw perception data collected by multi-sensors on an intelligent agent. This strategy often processes the homogenous data from the same type of sensors, such as cameras or LiDARs (Figure 2c). It has a higher accuracy of environment perception without information loss. But the method has a high processing cost and is very time-consuming, while processing a lot of raw data.

In summary, different sensors have merits and limitations in data collection. The proper integration of multi-source data from different sensors can enhance the perception accuracy of ICVs in complex weather conditions. Additionally, the architectures of multi-sensor data fusion methods are summarized as the centralized architecture, the distributed architecture, and the hybrid architectures based on the allocation of computing resources for fusion computation within intelligent perception agents. Fusion strategies can be classified into three levels: target-level fusion, feature-level fusion, and data-level fusion. Table 3 illustrates the merits and limitations of the three data fusion strategies [100]. However, it is noteworthy that when detected objects are far away from sensors, the perception accuracy declines greatly. This is attributed to the loss of texture and color features in the image and the reduced availability of sparse point cloud data.

## 4. Vehicle–Infrastructure Cooperative Perception Method

The environment perception of ICV includes autonomous environment perception and cooperative environment perception. Autonomous environment perception of ICVs faces two primary challenges in adverse weather conditions: insufficient perception information and the limited computing capabilities. The development of 5G networks and Vehicle to Everything (V2X) communication technology [101,102] has made it possible for cooperative environment perception to utilize the perception information from roadside infrastructures or other ICVs. The sufficient fusion information in adverse weather conditions can enhance environment perception accuracy, and broaden the range of ICV perception. And roadside computing units also contribute to computing resources to help ICVs process multisource perception data and achieve the optimal distribution of perception information. The main research in vehicle–infrastructure cooperative perception methods includes multisource information fusion and information distribution strategies between Vehicle to Vehicle (V2V) [103] and Vehicle to Infrastructure (V2I) [104] to avoid channel congestion and optimize the distribution of perception information.

Qiu et al. [103] and Chen et al. [89] proposed a cooperative perception system to broaden the field-of-view of ICVs. The system directly distributed raw visual information with other ICVs. Schiegg et al. [105] and Shan et al. [106] demonstrated that the cooperative perception among ICVs enhances the environment perception capability of ICV, and increases the correctness of autonomous driving decisions. Cui et al. [107] and Zhao et al. [108] demonstrated the advantages of cooperative perception in transportation systems, using LiDAR data from roadside units to validate the broadcasting mechanism for object detection results in V2I. Ma et al. [109] merged detection information from roadside cameras, which were installed in multiple areas along a road. The study used the fusion information to create a global semantic description, assisting ICVs in making decisions in complex traffic environments. Yu et al. [110] employed perception data from ICVs and roadside infrastructures to construct the DAIR-V2X dataset. This large-scale, multi-source, multi-view dataset supported vehicle–infrastructure cooperative perception and decision research based on computer vision. Chao et al. [111] utilized object detection results and location data obtained from roadside infrastructures to assist the self-driving cars in solving perceived blind zones. The information included point cloud data after clustering, and visual semantic information.

### 4.1. Information Fusion Strategies in Cooperative Perception

According to the different scales of information fusion, fusion strategies in V2V or V2I cooperation perception can be divided into early information cooperation, late information cooperation, medium-term information cooperation, and hybrid feature fusion cooperation.

**Figure 3:**
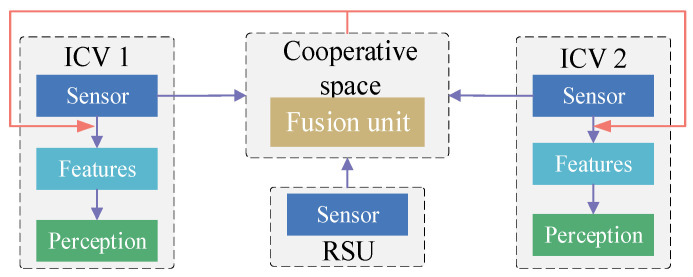
Early information cooperation framework of vehicle–infrastructure cooperative perception.

Early information cooperation [89] directly distributes the raw perception data between V2V and V2I. And the data are projected into the same coordinate space. Each vehicle in perceptive areas can access all the data, but the strategy requires a large transmission bandwidth (Figure 3). Arnold et al. [92] used an early cooperative model to achieve vehicle–infrastructure cooperative perception. The results demonstrated that a higher detection accuracy could be obtained using a mass of raw perception information, but the abundance of information tends to overload the communication network. Li et al. [112] constructed Distilled Collaboration Graph Network (DiscoNet) with intermediate feature mapping based on a knowledge distillation algorithm to reduce the amount of information transferred under the limited communication bandwidth. The method utilized the teacher model trained in early collaboration to obtain model parameters, and these parameters were utilized in the student model. The student model was trained with intermediate collaboration features.

**Figure 4:**
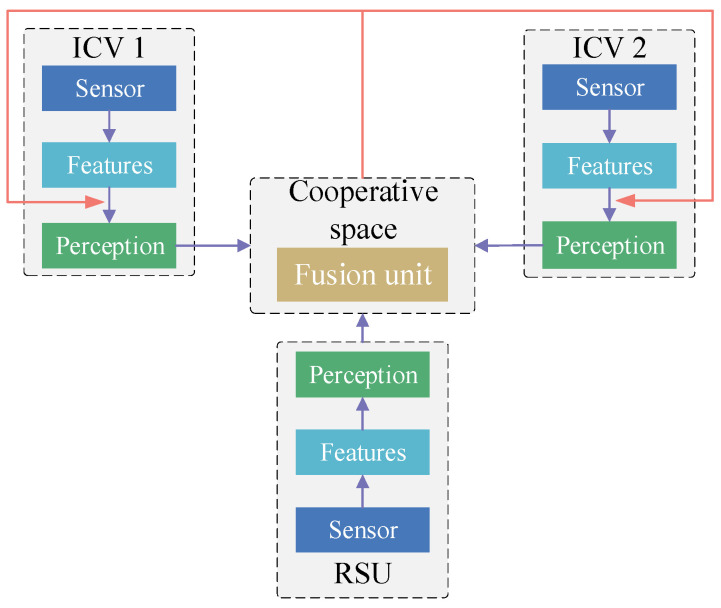
Late information cooperation framework of vehicle–infrastructure cooperative perception.

Late information cooperation [48] inputs perception results from ICVs and roadside units (e.g., object detection boxes, confidence scores) into a unified perception space to achieve environment perception. This information distribution strategy does not need to access the underlying detection network of other perception devices. Thereby, it reduces the bandwidth pressure of information transmission. (Figure 4). Zhao et al. [113] utilized lane detection tags from roadside infrastructures and ICVs to compute uncertainty using the Dempster–Shafer theory. Shangguan et al. [114] integrated a multi-target tracking method [115] and a voxel clustering algorithm to obtain the perception results for the environment around the target vehicle using on-board data. To achieve an accurate vehicle trajectory, the perception results from the target vehicle were merged with those from roadside units and other ICVs. Vadivelu et al. [116] utilized an end-to-end learned neural inference layer to estimate state errors. These state errors were used to reduce the positioning noise in a vehicle’s status perception data. Mo et al. [117] constructed a two-stage Kalman filter in late information cooperation to remove anomalous cooperation information generated during roadside infrastructure failure.

**Figure 5:**
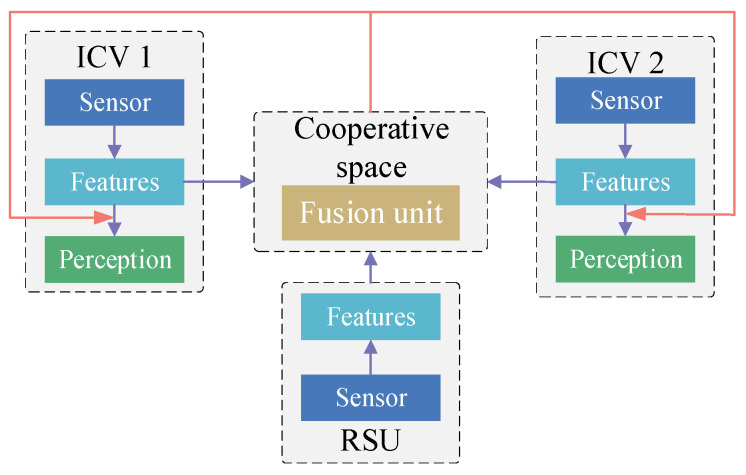
Medium-term information cooperation framework of vehicle–infrastructure cooperative perception.

Medium-term information cooperation [118,119] requires each perception unit to generate intermediate features using a prediction model. The intermediate features are compressed at RSU before distribution to target vehicles. Target vehicles need to decode the compressed features when using them to perceive the environment. The information fusion method needs a lower communication channel compared to the early information cooperation, and obtains more information from other perception units compared to the late information cooperation (Figure 5). However, the challenge of this method lies in compressing feature data without data loss, and accurately extracting distributed features on target vehicles. Emad et al. [31,120] conducted extensive research on data compression and optimal extraction of shared information. They used a decentralized shared data alignment method to obtain the fusion features. Sridhar et al. [121] employed cooperative relative localization and a high-definition map to match visual perception results from multiple ICVs in perception space. Wang et al. [122] utilized a Vehicle to Vehicle Network (V2VNet) to extract intermediate features from an object detection backbone. And a graph neural network was used to aggregated features from other ICVs. Liu et al. [123] used the 3D projection model to obtain feature points among ICVs, and those feature points were used to estimate geometric transformation parameters. These parameters were utilized in depth mapping transformation to integrate feature information.

**Figure 6:**
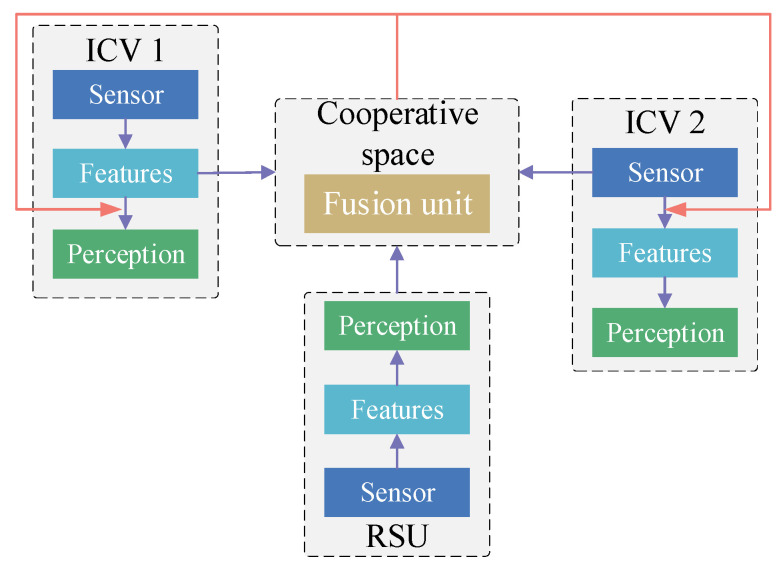
Hybrid information cooperation framework of vehicle–infrastructure cooperative perception.

Hybrid information cooperation integrates two or more cooperative perception strategies to improve the perceptive precision and reduce communication delay during cooperative perception (Figure 6). Arnold et al. [92] adaptably chose to share high-level information (perception results) or low-level information (raw perception data) according to the density and visibility of object information obtained from sensors. Glaser et al. [124] utilized neural networks to learn the corresponding relationship among perception information from multiple ICVs and discarded pose information from other intelligent agents. Cui et al. [125] constructed an end-to-end feature transformation learning model to achieve cooperative perception in V2V. The method included image information processing, point cloud coding, and multi-source information fusion in complex environments. Xu et al. [119] constructed an adaptive V2X transform architecture to extract parallelly local features and merge adaptively global spatial features among multiple heterogeneous intelligent agents. The architecture integrated a heterogeneous multi-agent attention module and a multi-scale window attention module to solve asynchronous information distribution, positioning errors, and communication heterogeneity.

Early information cooperation, late information cooperation, and medium-term information cooperation are often used in VICS research. This paper summarizes and analyzes some representative studies in each information fusion strategy, and Table 4 shows the key research points, merits and limitations of these studies.

### 4.2. Information Fusion Methods in Cooperative Perception

Multi-source information fusion is the core of vehicle–infrastructure cooperative systems. Information Fusion Methods in cooperative perception must process a lot of heterogeneous data from various perception units and achieve the representation of multi-source information in the same time and space.

It is crucial to employ some methods that select optimal fusion information in the cooperative space, which contains a large variety of information types from different perceptive units. This optimal information selection can enhance the target vehicle’s accurate perception of the environment, including object detection, trajectory tracking and so on. Yu et al. [110] constructed a 3D target detection framework based on time compensation to avoid time asynchrony between vehicle–infrastructure cooperative localization and the 3D results of object detection. It could reduce the data transmission cost. Neubeck et al. [44] utilized Non-maximum Suppression (NMS) to aggregate bounding box suggestions from neighboring intelligent agents, and used the confidence scores to merge multiple candidate boxes.

Sukhbaatar et al. [126] constructed a CommNet information fusion model, using averaging operator to merge the information from vehicles and roadside infrastructures. Hoshen et al. [127] introduced a Vertex Attention Interaction Network (VAIN). The network utilized an attentional architecture to learn multi-agent shared information and provide prediction results with small computation budgets. Vaswani et al. [128] integrated Transformer with feed-forward stacking layers and a self-attention mechanism with multi-detector heads. The method could achieve interaction at a long distance between multiple intelligent agents. Jiang et al. [129] and Liu et al. [130] utilized matrix elements with border weighting to represent the attention in a specific spatial area among the intelligent agents. The method adaptively calculated the spatial information of target vehicles and positional relationships among perceptive units. Chen et al. [91] constructed an F-Cooper model based on the maximum output mechanism. The model could select the optimal shared features from 3D point cloud. Wang et al. [122] utilized a V2VNet network to learn spatial features based on map fusion. And the map fusion included position and state information among multiple intelligent agents. The network used a spatial message-passing mechanism for joint reasoning in cooperative space. Cheng et al. [131] used an extended Kalman filter to compensate for the delay in position and relative measuring distance of multiple intelligent agents after the temporal and spatial alignment of the perception information. Xu et al. [118] constructed the first large-scale Open Dataset for Perception with V2V communication (OPV2V), based on the data from a single detection head of ICVs or roadside infrastructures. To enhance the accuracy and adaptability of information fusion among multiple intelligent agents, many fusion methods have integrated the self-attention mechanism into multiple layers of the fusion network, such as Swin Transformer [132], Cross-Shaped Window (CSWin) [133], and Twins [134]. They utilized a hierarchical structure to increase the amount of acceptable information and incorporate the longer dependency terms. However, these studies mainly used the data with the same data structure to construct fusion models. Further research is needed for methods that can process heterogeneous data fusion or eliminate the data noise generated by adverse weather conditions.

After integrating perception information from multiple intelligent agents, the representation of the fusion information in perception space can improve the target vehicle’s accuracy in environment perception. Noh et al. [135] integrated cooperative perception data with high-precision maps to obtain driving advice in adverse weather conditions or unexpected traffic events, which was sent to the target vehicle by roadside infrastructure within communication range. Dosovitskiy et al. [136] constructed a vision transformer (ViT) to achieve global interactions in perceptive space. The ViT model integrated full self-attention mechanisms and image patching. However, the method’s calculations are highly complex and may not be suitable for remote sequences and high-resolution images. Xu et al. [137] proposed a sparse vision transformer to generate BEV segmentation maps in cooperative perception space. The transformer used fusion axial attention modules to search local or global spatial information from different ICVs and different visual sensors in the perceptive space. Liu et al. [138] proposed a BEVFusion method, which converted image perception features to BEV. The method employed convolutional layers to merge the visual feature with LiDAR BEV features.

### 4.3. Information Sharing Methods in Cooperative Perception

Communication technology has advanced significantly, but it is difficult for OBU and RSU to compute a mass of multi-source heterogeneous information in a perceptive space due to the limited calculation resources in these devices. If information sharing methods fail to distribute the optimal cooperative information to target vehicles, they cause communication transmission delays, information packet loss, and channel congestion.

Numerous studies have focused on optimal information distribution methods in communication networks by reducing the information redundancy, selecting the best sharing frequency, and the optimal data formats. Liang et al. [45] and He et al. [139] used graph techniques to achieve the optimal distribution in vehicle–infrastructure cooperative systems. Each V2V link between ICVs was regarded as a node of the graph, which was used to calculate the distribution weights. Allig et al. [140] transformed non-predictive sender states and discarded motion compensation in the perceptive space for time and space synchronization to avoid communication transmission delays. Higuchi et al. [141] used a prediction model to predict the importance of a Cooperative Perception Message (CPM). And the predicted result was used to decide whether to send the CPM driving strategy to a target ICV. The method was able to reduce the redundant information in communication channels. Talak et al. [142] selected ROI data from various multi-sensor data and distributed them to target vehicles based on the optimal update frequency of CPMs. The method can reduce channel congestion and avoid information packet loss. Li et al. [112] integrated a knowledge distillation model with an attention mechanism to reduce the amount of sharing data features. The attention mechanism used masks to reflect intelligent agents’ perceptive information in a spatial region. Vadivelu et al. [116] utilized a positioning error regression module to correct noise errors in the received positioning information. Chen et al. [143] used a confidence calibrator with double boundary scaling to reduce confidence score bias due to heterogeneous perceptive models in different intelligent agents. The multi-agent perception framework was not influenced by the structure of perception models in different perception units, and used aggregation algorithm to ensure the consistency of confidence values for adjacent detection boxes in the same space.

In summary, many studies have carried out a lot of experiments to demonstrate the advantages of vehicle–infrastructure cooperative perception based on simulation data or actual traffic data. The approach can enhance the perception range and precision of ICVs in blind zones by merging the cooperation information. These studies mainly researched some optimization algorithms to choose the optimal information from redundant data, optimal information compression, transmission, and fusion mode within limited communication channels or communication delays. Most of them use homogeneous data (such as images or LiDAR points) and the same feature extraction mode or object detection methods to obtain homogeneous cooperation information. They do not consider the applicability of the methods when adverse weather causes negative factors in raw data or features during early cooperation and medium-term cooperation. Further research needs to focus on an adaptive calibration method for perception results obtained from different object detection modes during late information cooperation.

## 5. Discussion and Outlooks

In order to improve perception precision and extend the application scenarios of ICVs, many studies have researched pre-processing methods, multi-sensor fusion methods, and cooperation perception methods.

The pre-processing methods aim to eliminate the negative effects of raw perception data, caused by adverse weather conditions, such as fog, rain, and snow. The paper mainly summarizes LiDAR point denoising methods using statistical filtering, neighborhood filtering, projection filtering, and voxel filtering. For image enhancement, methods like semantic segmentation, depth estimation [18], low-pass filters [19], and multi-scale dense networks [77] are discussed. These data, after removing outliers and noisy data, are important to perform early information cooperation in VICS and to use to extract target features in object detection or medium-term information cooperation. These methods have shown good processing results in mist, light rain, and light snow, but the preprocessing results in dense fog, heavy rain and snow perform poorly.

Numerous multi-sensor fusion methods have been explored with homogeneous data (e.g., image and image; LiDAR point and LiDAR point) and heterogeneous data (e.g., image and LiDAR point) to obtain more abundant information and reduce the uncertainty of a single sensor in adverse weather conditions. These methods utilize Kalman filter-based fusion estimation, Bayesian-based distributed fusion methods, and neural network-based methods (e.g., CNN [23], GAN [21], and RadarNet [23]) to obtain fusion data, features, and perception results. These data are used in target detection algorithms to obtain more accuracy classification results. These results can be employed in late information cooperation and prompt ICVs to reach L3 and L4 with high-definition maps in some circumstances. However, each sensor has a limited perception range. It leads to a loss of texture and color features in images and has sparse LiDAR points when detected objects are far away from sensors. And the perception accuracy declines greatly.

According to those studies using redundancy information from the adjacent frames or multi-sensors on ICVs to preprocess raw data and enhance perception precision in adverse weather conditions, it was found that VICS may represent a promising approach to overcome computing bottlenecks, address insufficient perception capabilities of ICVs, and promote Level 5 vehicles. This paper analyzed the merits and limitations of three information fusion strategies and summarizes multi-source information fusion methods using NMS [29], VAIN [127] and Swin Transformer [132]. And the optimal information distribution methods are discussed under limitation communication networks, such as graph techniques [139], knowledge distillation models [112] between ICVs and roadside infrastructures. At present, these methods mainly use homogeneous data, the same feature extraction modes or object detection methods to obtain homogeneous cooperation information. And they are tested in a junction with good weather or a road with blind areas.

After discussing these studies on data preprocessing methods in adverse weather conditions, multi-sensor fusion methods based on ICVs, and cooperative perception methods between ICVs and roadside units, there are still many challenges for achieving commercial applications of higher-level autonomous vehicles in any circumstances. In the future, the cooperative perception method in adverse weather conditions should focus on the following aspects:

(1) Raw data preprocessing in adverse weather conditions is mainly to judge, denoise and repair by identifying obvious features between normal data and noisy data. Future work can focus on using multi-scale information by integrating depth and semantic information to obtain more features of rain and snow, and utilizing the data difference based on early information cooperation strategy to filter the noisy data.
(2) The cooperation perceptive networks only deal with homogeneous data, features and detection results from the same extraction mode or object detection methods. Future work can explore an adaptive calibration method or construct a uniform standard specification to standardize the format of detection results and extracted features obtained from different data types and algorithms in early- or medium-term cooperation.
(3) If we just use a kind of information cooperation strategy to achieve cooperation perception, it is hard to balance the accuracy and computing speed of an artificial neural network in any conditions. Future work can innovate a hybrid information cooperation method to adaptively share extracted features or raw perception data based on motion prediction results and importance scores. These scores are determined by predicting the importance of a CPM.
(4) Some cooperation perception networks often integrate various modules into the backbone network to obtain a higher accuracy. These modules make the networks large and complex, which need high computing units. Further research needs to focus on a lightweight deep learning network by utilizing a powerful learning ability of artificial intelligence in data preprocessing, object detection, and cooperative perception.
(5) The current deep learning algorithms mainly rely on the specific features and labeled data for environment perception. Different traffic scenarios require to build different datasets and a large number of labeled data. It severely limits the generality and migration of the deep learning algorithms. Future efforts focus on constructing networks with unsupervised learning, self-supervised learning and autoencoders.

## 6. Conclusions

Intelligent connected vehicles operating at a higher automated level face significant technical challenges and limitations in environmental perception, especially in adverse weather conditions. This paper reviews several current issues in ICVs under adverse weather conditions and summarizes the preprocessing methods for raw images and LiDAR points, as well as environment perception based on multi-sensor fusion. But these methods still have some shortages, such as low perception precision at a distance, and insufficient noise removal. The vehicle–infrastructure Cooperative System emerges as a promising approach to compensate the insufficient perception information by utilizing V2V and V2I communication. This approach reduces the limitations of on-board computing units by utilizing sufficient roadside computing resources. This paper mainly summarizes and analyzes information fusion strategies and cooperation information sharing methods in a cooperative perception environment. With the rapid development of wireless communication technologies such as LTE-V2X and DSRC, the VICS will play an important role in the future of fully automated vehicles. However, there are still many challenges in the practical application of the cooperation perception methods. The challenges include the handling of heterogeneous cooperation information, efficient reduction in redundant cooperation data, and the optimal distribution of information to ICVs within the constraints of communication bandwidth. This paper not only discusses the major contributions and the limitation of existing models, but also proposes future research directions to overcome these challenges.

## Figures and Tables

**Table 1:** The taxonomy of intelligent connected vehicles.

Level	Name	Lateral and Longitudinal Vehicle Motion Control	Object and Event Detection and Response	Dynamic Driving Fallback	Working Conditions
Level 0 vehicle (L0)	Fully manual driving vehicle	Driver	Driver	Driver	All circumstances
Level 1 vehicle (L1)	Partial driver assistance vehicle	Driver and autonomous driving system	Driver	Driver	Partial circumstances
Level 2 vehicle (L2)	Combined driver assistance vehicle	Autonomous driving system	Driver	Driver	Partial circumstances
Level 3 vehicle (L3)	Conditionally automated driving vehicle	Autonomous driving system	Autonomous driving system	Driver	Partial circumstances
Level 4 vehicle (L4)	Highly automated vehicle	Autonomous driving system	Autonomous driving system	Autonomous driving system	Partial circumstances
Level 5 vehicle (L5)	Fully automated vehicle	Autonomous driving system	Autonomous driving system	Autonomous driving system	All circumstances

**Table 2:** The five stages of development of VICS.

Name	Major Technology	Construction Content	Service Subject
VICS1.0	Physical information and optical technology	Setting up signs and linear guidance facilities on the road. And reflectors are mainly used to solve the blind area problem for drivers in the curve segment of roads and intersections.	Ordinary vehicles
VICS2.0	Road variable speed control and information broadcast technology	Implementing variable speed limit signs and speed control system to instruct drivers, achieving uniform speed changes and avoiding tail-end collision accidents.	Ordinary vehicles
VICS3.0	Active safety warning technology	Installing coil detectors, microwave detectors, video cameras, geomagnetic detectors and LED screens to solve the problem of blind areas on curve roads and adverse weather conditions.	Ordinary vehicles
VICS4.0	Internet of things technology	Setting up electronic toll collection system, active luminous traffic signs, and using millimeter wave radar or machine vision to establish danger warning system, etc.	ICVs
VICS5.0	C-V2X communication technology (DSRC, LTE-V2X, 5G-V2X)	Constructing intelligent signal controllers, high-definition maps, cloud platforms, edge computing units to promote the innovation and application of autonomous driving technology, and using LiDAR sensors to obtain more information.	ICVs

**Table 3:** Summary and analysis of the three data fusion strategies.

Fusion Strategy	Merit	Limitation	Methods
Target-level fusion	Applied to a variety of sensors, low computation, high reliability and fault-tolerant	Low detection accuracy, high false positive rate, high preprocessing difficulty, maximum information loss	Artificial neural network, Bayes estimation, Dempster/Shafer (D-S) evidential reasoning,
Feature-level fusion	Data compression for real-time processing, the balance between detection accuracy and information loss	Heterogenous data preprocessing before fusion	Cluster analysis, artificial neural network, probability statistics and fuzzy logic reasoning
Data-level fusion	Abundant data, low preprocessing difficulty and best classification performance	Poor real-time performance, huge volume of data, long processing time, high processing cost	Weighted mean, Kalman filter, wavelet transform and principal component analysis (PCA) transform.

**Table 4:** Summary and analysis of information fusion Strategies.

References	Fusion Scheme	Key Research Points	Findings	Merit	Limitation
Arnold et al. [92]	Early cooperative fusion	Combining different point clouds from multiple spatially diverse sensing points and using the fusion data to perform 3D object detection.	The result shows more than 95% of the ground-truth objects are detected with precision above 95%.	Detection accuracy is high.	The communication bandwidth is cost highly due to a lot of raw data need to be transferred.
Li et al. [112]	Early cooperative fusion	Constructing a teacher–student framework with a novel distilled collaboration graph and a matrix-valued edge weight.	The average precisions are 60.3% at IoU = 0.5 and 53.9% at IoU = 0.7 separately, compared with 56.8% and 50.7% in the V2Vnet.	Achieving a better performance–bandwidth trade-off and detecting more objects.	
Shangguan et al. [114]	Late cooperative fusion	The fusion status of surroundings obtained by a Lidar-only multiple-object tracking method is used to generate the trajectories of target vehicles with the preliminary tracking result.	The method has a better performance especially when the Lidar is limited or the V2V communication is failed.	Improving the accuracy of object tracking and expanding the vehicle perception range.	Different real external environment is not considered, such as the partial equipment failure, the mixed traffic conditions, and poor cooperative information has a negative effect on the perceptual accuracy.
Mo et al. [117]	Late cooperative fusion	The traditional Kalman Filter is used to obtain position information when the roadside fails, and the state information helps target vehicles improve the average positioning accuracy.	The average positioning accuracy from vehicle infrastructure cooperative perception is 18% higher than vehicle-only perception.	The fusion framework provides CADS methods and systems for coordinating.	
Emad et al. [31,120]	Medium-term cooperative fusion	Grids of down-sampled feature data are distributed to increase detective performance, and an encoder/decoder bank is deployed to disentangle the communication bandwidth limitation.	The detective accuracy of pedestrians is 6% higher than translation MOD-Alignment method.	Average precision outperforms feature sharing cooperative object detection method.	If the method fails to compress feature data well or extract distributed features accurately, the perceptive precision and the use of communication bandwidth will be poor.
Wang et al. [122]	Medium-term cooperative fusion	A variational image compression algorithm is used to compress intermediate representations, and a convolutional network is used to learn the representations with the help of a learned hyperprior.	The result is 88.6% of average detection precision at IoU = 0.7, 0.79 m error at 3.0 s prediction, and 2.63 trajectory collision rate.	Achieving the best balance between accuracy improvements and bandwidth requirements.	
